# Environmental and occupational respiratory diseases – 1036. Preliminary audit of the allergy clinic and spectrum of aeroallergen sensitivity in allergic rhinitis

**DOI:** 10.1186/1939-4551-6-S1-P35

**Published:** 2013-04-23

**Authors:** MS Soumya

**Affiliations:** 1 St. John's Medical College Hospital, Bangalore, India

## Background

Allergic rhinitis (AR) is a heterogeneous disorder that despite its high prevalence is often undiagnosed.

The quality of life is significantly impaired in subjects with allergic rhinitis, but can be improved by treatment . However, the condition may frequently be trivialised (by the patient) and/or unrecognised (by the physician), resulting in the inadequate control of symptoms.

Many causative agents have been linked to AR including pollens, molds, dust mites, and animal dander. Aeroallergens play a major role in the pathogenesis of allergic diseases, particularly rhinitis and asthma.

## Objectives

1. To analyse the trends and patterns in patients with allergic rhinitis who visited our clinic.

2. To get baseline data regarding the spectrum of allergens.

3. To adapt appropriate treatment protocols.

4. To plan further studies.

5. To study the skin sensitivity to various allergens by skin prick test in patients of nasobronchial allergy.

## Methods

We conducted a clinical audit of the patients evaluated in the allergy clinic Jan 2011 – Jan 2012.

Study design: Cross-sectional observational study.

The patients were evaluated with a detailed history, anterior rhinoscopy and nasal endoscopy. Investigations including Absolute eosinophil count, Total serum IgE, Skin prick test and nasal smear for eosinophilia were performed. All these details were recorded in a proforma.

A panel of 22 allergens were tested which were broadly divided into 6 groups namely: fungi, pollen, dust, animal epithelia, dust mite and insects.

We present here the results of our annual audit and the allergen sensitivity pattern.

## Results

The skin prick positivity was highest among fungi (29.86%), followed by pollen (23.61%), dust (21.53%), animal epithelia (11.11%), dust mite (10.42%) and insects (3.5%). Overall the most common allergen was found to be Parthenium hysterophorus (44.9%).

## Conclusions

The present study serves as a baseline for selection of panel of aeroallergens for skin prick tests and also depicts the allergy pattern and trends in urban India. This study will further be corroborated by establishing a pollen calendar specific for this region. Allergen avoidance and choice of allergens for immunotherapy will be planned on this basis.

